# Prevalence of overweight in 2 to 17 year-old children and adolescents whose parents live separately: a Nordic cross-sectional study

**DOI:** 10.1186/1471-2458-14-1216

**Published:** 2014-11-25

**Authors:** Lena Hohwü, Mika Gissler, Agneta Sjöberg, Anna M Biehl, Alfgeir L Kristjansson, Carsten Obel

**Affiliations:** Section for General Practice, Department of Public Health, Aarhus University, Bartholins Allé 2, 8000 Aarhus C, Denmark; THL National Institute for Health and Welfare, PO Box 3000271, Helsinki, Finland; Department of Food and Nutrition, and Sport Science, University of Gothenburg, Post Box 300405, 30 Gothenburg, Sweden; Division of Epidemiology, Norwegian Institute of Public Health, PO Box 4404, 0403 Nydalen, Oslo, Norway; School of Public Health, West Virginia University, PO Box 9190, Morgantown, WV 26506-9190 USA; NHV Nordic School of Public Health, Gothenburg, Sweden; Icelandic Centre for Social Research and Analysis, Reykjavik University, Reykjavik, Iceland

**Keywords:** Cohabitation, Divorce, Body mass index, Overweight, Children, Nordic

## Abstract

**Background:**

Comparative data of parental separation and childhood overweight has not been available before across the Nordic countries. The aim of this study was to examine the within-country prevalence and association between parental cohabitation and overweight in Nordic children.

**Methods:**

A cross-sectional survey of 2-17-year-old children was conducted in 2011, titled: “NordChild”. A random sample of 3,200 parents in each of the Nordic countries (Denmark, Finland, Iceland, Norway, and Sweden were invited to participate in the study with parents of 6,609 children accepting to give answers about their children’s health and welfare including information on height and weight of each child and parental cohabitation (response rate 41.5%). The group differences in prevalence and adjusted odds ratio (OR) for overweight, with corresponding 95% confidence intervals (CI) were performed in children whose parents lived separately. Additionally, a missing data analysis was performed to determine whether the adjusted estimates might result from confounding or selection bias.

**Results:**

A significant difference was observed in Iceland between children whose parents live separately compared to those who live with both parents (difference: 9.4%, 95% CI: 2.8; 15.9) but no such difference was observed in Denmark, Finland, Norway and Sweden. No significant odds of overweight were observed in children whose parents lived separately compared to children in normal weight at the time of study; Denmark: OR 1.03 (95% CI: 0.42; 2.53), Finland: OR 1.27 (95% CI: 0.74; 2.20), Iceland: OR 1.50 (95% CI: 0.79; 2.84), Norway: OR 1.46 (95% CI: 0.81; 2.62), and Sweden: 1.07 (95% CI: 0.61; 1.86). The missing data analysis indicated that the findings in Norway, Finland and Iceland were partly observed due to selection effects, whereas the adjustment in Denmark was due to confounding. The crude OR for overweight was higher in the 2-9-year-old group than in the 10-17-year-old group whose parents lived separately in Iceland, Norway and Sweden.

**Conclusions:**

No association between parental cohabitation and overweight in Nordic children was found. Our finding of greater prevalence of overweight in Icelandic children whose parents live separately may be an indication that the welfare system in Iceland is separating from the other Nordic countries.

## Background

The prevalence of overweight in children and adolescents has increased greatly in most European countries during the last 30 years [1, 2]. This applies also to the Nordic countries [3–7], although recent evidence suggests that the prevalence rates of overweight may be levelling off in some of them [3, 6, 8]. By examining the association of exposure of psychological stress early in life and risk of overweight, we have earlier found that schoolchildren born of mothers who experienced loss of a close relative during the prenatal time period had an increased risk of being overweight [9]. Bereavement is a severe, but rare, stress exposure whereas parental separation or divorce is a broader and more common exposure that is not as severe [10]. A larger body of literature has showed a higher prevalence and increased risk of overweight in children whose parents have separated than in children whose parents are cohabiting [11–17]. However, some studies have showed that girls, but not boys, were more likely to be overweight if living with a single parent [5, 16]. These studies that have been performed in different countries have used different methods of data collection and analyses but studies that utilize comparative methods across countries are rare.

The populations in the Nordic countries are comparatively homogenous, but up to this point comparative data of family structure and children’s height and weight has not been available across the Nordic countries. The hypothesis of this study was that the prevalence of overweight in children whose parents lived separately compared to those living with both parents would be the same within each of the Nordic countries. Thus, we aimed to examine the prevalence of overweight and the association between parental cohabitation and overweight status in 2-17-year-old children and adolescents across the five Nordic countries.

## Method

### Sample and procedures

This cross-sectional study is based on the 2011 NordChild survey. The NordChild survey (“Health and welfare among children and young people in the Nordic countries”) was conducted in 1984, 1996 and 2011 in the five Nordic countries: Denmark, Finland, Iceland, Norway, and Sweden. The survey questionnaire included questions on the health of the children, and welfare of both the children and the parents. In each survey, information on changes in family structure since the birth of the child was collected, but in 2011 information of self-reported height and weight of each participating child was included for the first time. A total of 16,000 randomly selected parents of children between the ages of 2 to 17 years were invited to participate in the study across the five Nordic countries; one child per family was drawn from the population registries in each country. A paper questionnaire was mailed to the invited participants in each country; however in Denmark a mix-mode approach of paper and internet-based questionnaire was used including a small nonmonetary incentive. Of the 16,000 invited participants, 55 were excluded due to reluctance to participate or the child had turned 18 years after the original sample was selected, and before the questionnaire was mailed to the participants. Overall, 7,805 of 15,945 parents responded the questionnaire, with a response rate within each country as follows: Denmark 54.1%, Finland 48.1%, Iceland 47.5%, Norway 49.4%, and Sweden 45.7%. Further details of the 2011 NordChild data collection have been described elsewhere [18]. The study was approved by the Regional Ethical Board in Gothenburg (Dnr. 009–11, 22 February 2011).

In the present analyses additional 1,196 responses (15.3%) were excluded because of inadequate or invalid information regarding height, weight or parental cohabitation. The study population therefore consisted of a total of 6,609 2-17-year-old children that were born between 1994 and 2009 and where self-reported information of parental cohabitation and height and weight of each child was available (response rate for the present study: 41.5%).

### Measures

The information of parental cohabitation was based on the question “Has the living situation of the parents changed since the birth of the child”, and where the options “No change” or “Yes, separation/divorce” were combined with information on whether the mother and the father of the child lived at the same address as the child at time of the study. Children of parents who responded “yes” to changed living situation and whose mother or father did not live at the same address as the child comprised the study group (Separate), whereas children to parents who responded “no” to changed living situation and where both parents lived at the same address as the child comprised the comparison group (Cohabiting).

The body mass index (BMI) of the child was derived by questions of height and weight: “How tall is the child (measure without shoes and round up to full centimeter)” and “What is the child’s weight (weight the child without clothes and round up to whole kilogram)”. As the BMI in children differs by age and gender, and anthropometric and body composition measures change considerably in relation to their physiological growth, the dependent variable of overweight status including obesity was created by dichotomizing BMI according to the standardized age and gender-specific cut-off points defined by the International Obesity Task Force [19].

Information of potential confounders and covariates was limited in the study design. Included control variables were; gender (boys/girls), age of the child, calendar year of child’s birth (1994–2009), maternal and paternal educational levels (≤9 years, 10–11 years, ≥12 years), and maternal and paternal age (<35 years, 35–44 years, ≥45 years).

### Statistical analysis

A combined Nordic figure was not derived due to statistical interaction between countries and consequently stratification by country was performed. No gender-based differences were observed across the Nordic countries and therefore no results stratified by gender are reported. Significance of interaction between cohabitation and gender, and between cohabitation and country was tested using a likelihood-ratio test.

The descriptive characteristics between children of separated and cohabiting parents are tested using a Chi-square test. The overall prevalence of overweight, and prevalence of overweight and difference in proportion of prevalence between separate and cohabiting parents with corresponding 95% confidence intervals (CI) was estimated within each of the Nordic countries.

We used multiple logistic regression analysis to examine the association between parental cohabitation and overweight in the data and report crude and adjusted odds ratios (OR) with corresponding 95% CI. We entered the data using a stepwise approach as parental level of education has been shown to be associated with overweight in adolescents [5]: First we stratified only by calendar year of birth, and gender and age of the child. Second, by adding maternal educational level and age, and third, we added paternal educational level and age. Furthermore, we performed a missing data analysis to determine whether the adjusted estimates were a result of confounding or selection bias. The missing analysis was based only on the number of observations available in the last step of the stepwise approach and we used multiple logistic regression analysis to estimate another crude and stepwise adjusted OR. If the two estimated crude OR differed substantially, the adjusted estimates most likely were caused by selection effects.

A sub-analysis of OR was also performed, stratified by age groups, to examine whether the association was different in younger and older children. Within each of the Nordic countries, the age was divided into two equal sized age groups: 2-9-years and 10-17-years. Due to a limited number of participants within each age group a crude estimation was only performed.

A *p* value below 0.05 was considered statistically significant.

A written informed consent for participation in the study was not requested as it is not required in the Nordic countries for this kind of study.

Data analyses were conducted with STATA version 11.0, software (StataCorp LP, College Station, TX, USA).

## Results

The descriptive characteristics of the separate and cohabiting groups within each of the Nordic countries are presented in Table 1. The proportion of separate parents in our sample was 19.6% in Sweden, 16.9% in Finland, 16.8% in Norway, 15.9% in Iceland, and 14.5% in Denmark. The largest proportion of mothers with low levels of education was observed in Finland (26.4%) and Iceland (34.9%). This pattern was also observed for low level of paternal education, although the proportions were not as high as for the mothers (see Table 1).

**Table 1:** **Descriptive statistics for study variables in the 2011 NordChild survey by country (N = 6,609)**

	Denmark (n = 1,610)					Finland (n = 1,240)					Iceland (n = 1,263)					Norway (n = 1,279)					Sweden (n = 1,217)				
	Separate		Cohabiting			Separate		Cohabiting			Separate		Cohabiting			Separate		Cohabiting			Separate		Cohabiting		
	n	(%)	n	(%)	*P* ^*a*^	n	(%)	n	(%)	*P* ^*a*^	n	(%)	n	(%)	*P* ^*a*^	n	(%)	n	(%)	*P* ^*a*^	n	(%)	n	(%)	*P* ^*a*^
**Participants**	234	(14.5)	1,376	(85.5)		210	(16.9)	1,030	(83.1)		201	(15.9)	1,062	(84.1)		215	(16.8)	1,064	(83.2)		239	(19.6)	978	(80.4)	
**Gender**																									
boys	119	(15.0)	677	(85.1)		95	(15.9)	501	(84.1)		101	(15.4)	54	(84.6)		104	(16.4)	531	(83.6)		127	(20.3)	499	(79.7)	
girls	115	(14.1)	699	(85.9)	0.64	115	(17.9)	529	(82.1)	0.37	100	(16.5)	508	(83.6)	0.62	111	(17.2)	533	82.8)	0.68	112	(19.0)	479	(81.0)	0.56
**Age groups**																									
2-5 years	32	(8.2)	358	(91.8)		29	(10.0)	260	(90.0)		24	(8.3)	266	(91.7)		13	(4.7)	261	(95.3)		17	(6.1)	263	(93.9)	
6-9 years	53	(12.9)	358	(87.1)		51	(14.3)	306	(85.7)		42	(13.7)	265	(86.3)		51	(15.7)	274	(84.3)		45	(14.7)	262	(85.3)	
10-13 years	83	(20.0)	333	(80.0)		79	(23.6)	256	(76.4)		66	(20.1)	263	(79.9)		70	(19.5)	289	(80.5)		85	(25.9)	243	(74.1)	
14-17 years	66	(16.8)	327	(83.2)	<0.01	51	(19.7)	208	(80.3)	<0.01	69	(20.5)	268	(79.5)	<0.01	81	(25.2)	240	(74.8)	<0.01	92	(30.5)	210	(69.5)	<0.01
**Maternal age**																									
< 35 years	21	(9.9)	192	(90.1)		51	(19.8)	186	(80.2)		48	(16.6)	242	(83.4)		25	(12.0)	183	(88.0)		28	(14.8)	161	(85.2)	
35-44 years	113	(12.8)	771	(87.2)		95	(14.3)	571	(85.7)		85	(13.5)	544	(86.5)		106	(15.0)	603	(85.0)		118	(17.4)	560	(82.6)	
≥ 45 years	68	(17.5)	320	(82.5)	0.02	46	(16.7)	254	(83.3)	0.13	48	(16.0)	253	(84.0)	0.40	44	(16.1)	229	(83.9)	0.44	60	(20.8)	228	(79.2)	0.22
**Maternal educational level**																									
≤9 years	6	(12.0)	44	(88.0)		14	(26.4)	39	(73.6)		38	(20.7)	146	(79.4)		19	(19.4)	79	(80.6)		15	(34.9)	28	(65.1)	
10-11 years	48	(15.6)	260	(84.4)		39	(20.3)	153	(79.7)		13	(15.3)	72	(84.7)		13	(17.3)	62	(82.7)		32	(21.2)	119	(78.8)	
≥12 years	120	(12.0)	879	(88.0)	0.26	138	(14.8)	796	(85.2)	0.02	106	(12.6)	735	(87.4)	0.02	150	(14.8)	866	(85.2)	0.42	156	(16.6)	784	(83.4)	<0.01
**Paternal age**																									
< 35 years	7	(5.5)	121	(94.5)		18	(13.1)	119	(86.9)		23	(12.7)	158	(87.3)		14	(12.3)	100	(87.7)		17	(15.6)	92	(84.4)	
35-44 years	40	(5.8)	652	(94.2)		54	(9.7)	55	(90.3)		44	(8.6)	469	(91.4)		54	(9.2)	535	(90.8)		63	(11.2)	499	(88.8)	
≥ 45 years	41	(7.8)	485	(92.2)	0.33	41	(9.7)	381	(90.3)	0.46	26	(6.5)	375	(93.5)	0.05	42	(10.3)	364	(89.7)	0.56	46	(11.5)	354	(88.5)	0.42
**Paternal educational level**																									
≤9 years	9	(6.6)	128	(93.4)		12	(15.4)	66	(84.6)		20	(11.0)	162	(89.0)		19	(15.6)	103	(84.4)		11	(12.1)	80	(87.9)	
10-11 years	26	(7.6)	318	(92.4)		47	(12.6)	327	(87.4)		6	(8.3)	66	(91.7)		13	(12.3)	93	(87.7)		31	(15.1)	174	(84.9)	
≥12 years	40	(5.4)	699	(94.6)	0.38	49	(7.7)	589	(92.3)	0.01	57	(7.7)	686	(92.3)	0.35	77	(9.0)	780	(91.0)	0.06	85	(11.1)	678	(88.9)	0.30

^a^P value, chi square test

Cohabiting: Parents of the child lived together.

Separate: Parents of the child lived separately.

The overall prevalence of overweight in 2-17-years-old children, and prevalence of overweight and difference in prevalence proportion between the separate and cohabiting groups are shown in Table 2. The difference in proportion of overweight children between the separate and cohabiting groups was only statistically significant in Iceland where the largest difference was observed or 9.4 percentage points (95% CI: 2.8, 15.9). The overall prevalence was similar in Finland, Norway and Sweden, whereas the prevalence of 11.2% in Denmark and 19.0% in Iceland differed from the other three countries.

**Figure 1:**
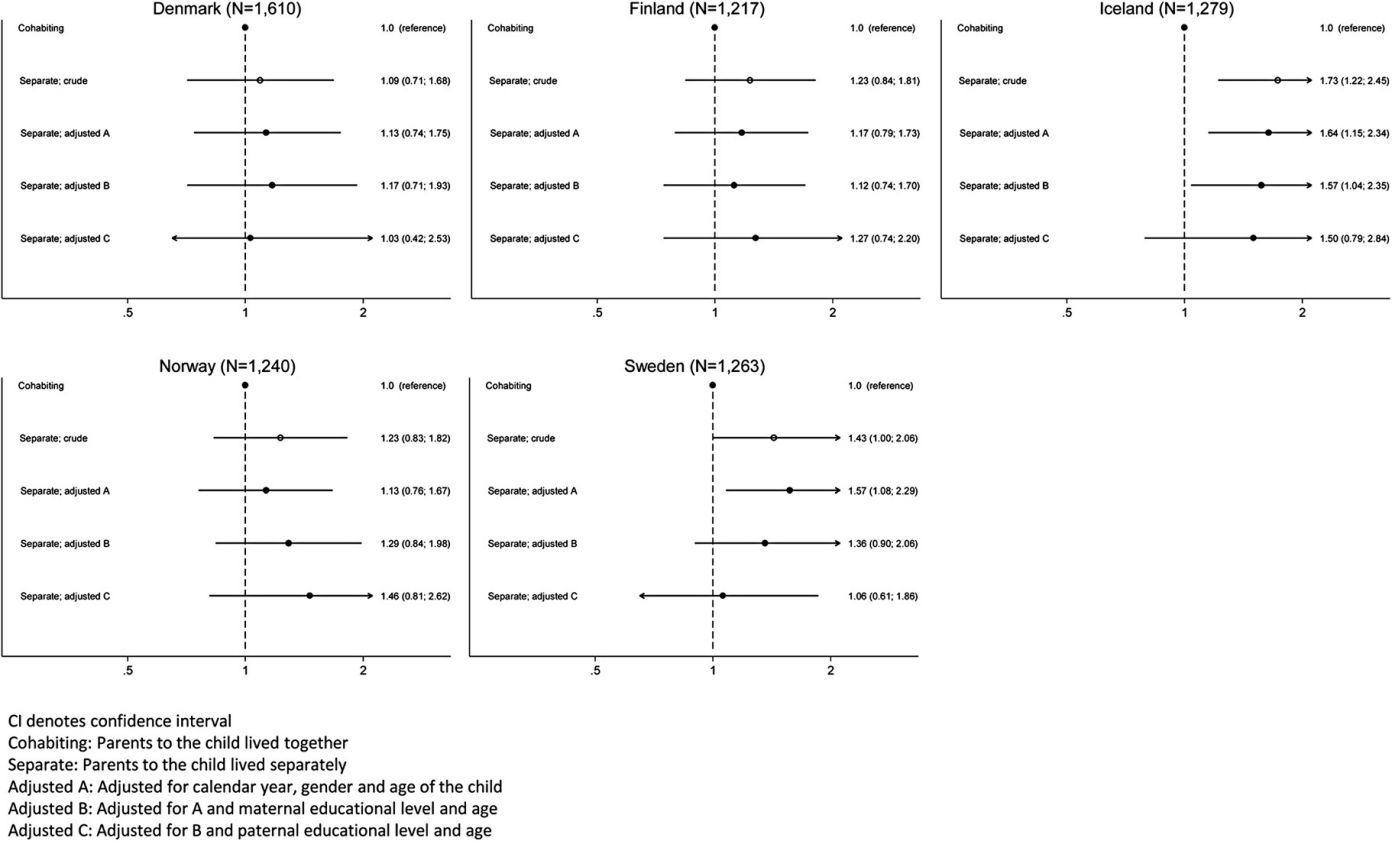
**The odds ratio (95% CI) for overweight in 2-17-year-old children whose parents lived separately at the time of study, stratified by country.**

The crude and stepwise models of adjusted ORs are shown in Figure 1. In short, within each country, we found no statistically significant association for overweight in children whose parents lived separately compared to children whose parents lived together at the time of study in the fully adjusted models; Denmark: OR 1.03 (95% CI: 0.42; 2.53), Finland: OR 1.27 (95% CI: 0.74; 2.20), Iceland: OR1.50 (95% CI: 0.79; 2.84) Norway: OR 1.46 (95% CI: 0.81; 2.62), and Sweden: OR1.07 (95% CI: 0.61; 1.86).

**Figure 2:**
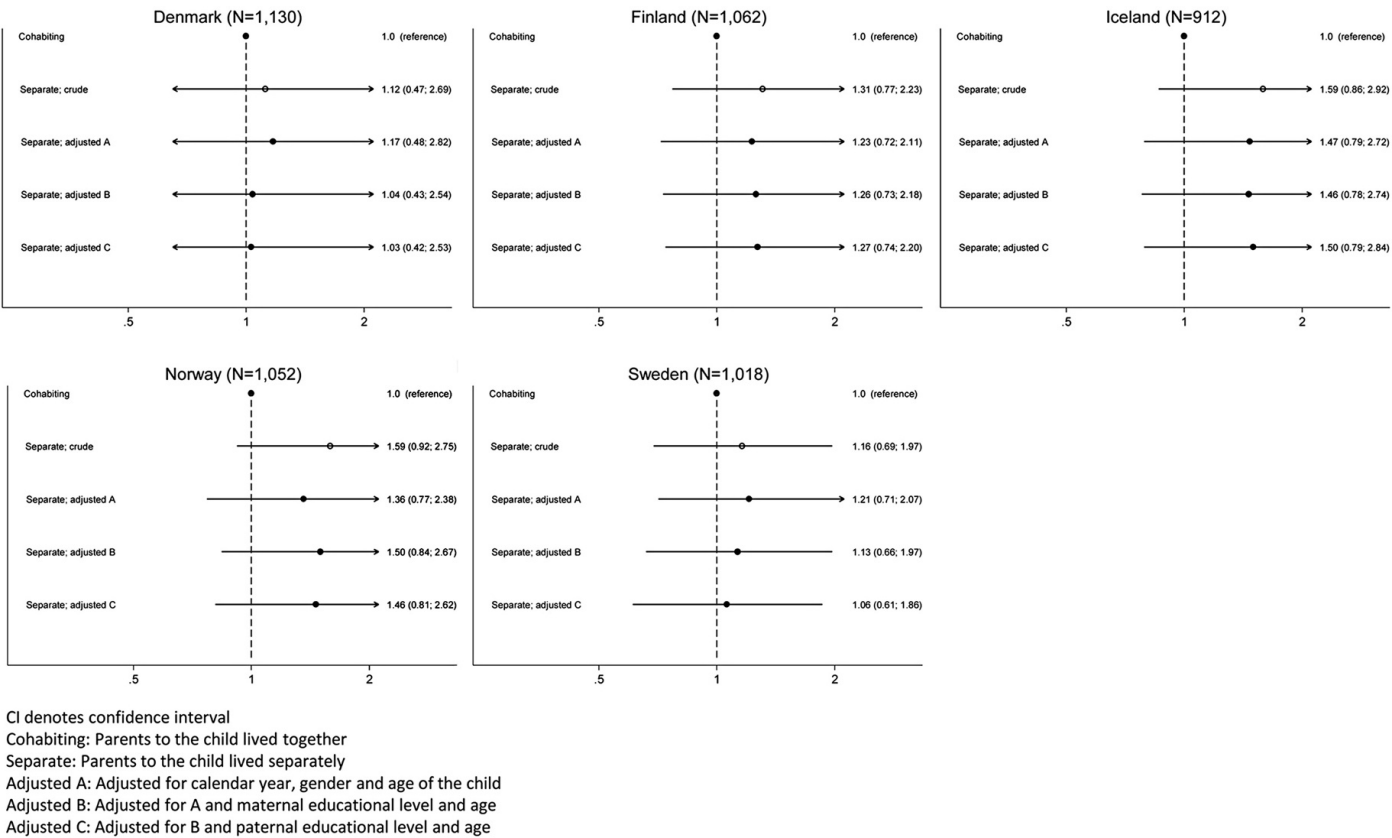
**Missing analysis based on observations included in the adjusted analysis only.** The odds ratio (95% CI) for overweight in 2-17-year-old children whose parents lived separately at the time of study, stratified by country.

The missing analysis of the association is shown in Figure 2. We found that the adjusted OR in Finland most likely was caused by selection as the two crude ORs differed: 1.73 versus 1.59, p = 0.03 (Figure 1 versus Figure 2). In Finland, Norway and Sweden the adjustment may partly have been due to selection; the crude ORs were 1.23 versus 1.31 (p = 0.23) for Finland, 1.23 versus 1.59 (p = 0.29) for Norway, and 1.43 versus 1.16 (p = 0.24) for Sweden (Figure 1 versus Figure 2). In Denmark, we found the adjustment most likely was due to confounding as the two crude ORs were similar; 1.09 versus 1.12, p = 0.15 (Figure 1 versus Figure 2).

**Table 2:** **The prevalence of overweight and difference in prevalence proportion in 2-17-year-old children whose parents lived separately at the time of study, stratified by country**

		Overweight				
	n	%	(95% CI)	Diff ^a^ %	(95% CI)	*P* ^b^
**Denmark**						
Overall	1,610	11.2	(9.6; 12.7)			
Cohabiting	152^c^	11.0	(9.4; 12.7)			
Separate	28^c^	12.0	(7.8; 16.1)	0.9	(-3.6; 5.4)	0.69
**Finland**						
Overall	1,240	16.5	(14.5; 18.6)			
Cohabiting	165^c^	16.0	(13.8; 18.3)			
Separate	40^c^	19.0	(13.7; 24.4)	3.0	(-2.7; 8.8)	0.30
**Iceland**						
Overall	1,263	19.0	(16.8; 21.2)			
Cohabiting	186^c^	17.5	(15.1; 19.7)			
Separate	54^c^	26.9	(20.7; 33.0)	9.4	(2.8; 15.9)	<0.01
**Norway**						
Overall	1,279	15.3	(13.3; 17.3)			
Cohabiting	158^c^	14.8	(12.7; 17.0)			
Separate	38^c^	17.8	(12.6; 22.8)	2.8	(-2.7; 8.4)	0.32
**Sweden**						
Overall	1,217	16.3	(14.2; 18.3)			
Cohabiting	149^c^	15.2	(13.0; 17.5)			
Separate	49^c^	20.5	(15.4; 25.6)	5.3	(-0.3; 10.9)	0.07

^a^Difference (prevalence proportion in Separate minus prevalence proportion in Cohabiting).

^b^P value, chi square test.

^c^n denotes the number of overweight children.

% denotes prevalence proportion.

CI denotes confidence interval.

Cohabiting: Parents of the child lived together.

Separate: Parents of the child lived separately.

Table 3 shows the crude analyses stratified by the two age groups 2-9-years and 10-17-years. Within each country, the crude OR for overweight was higher in the youngest than the oldest age group. For the 2-9-years-old children, the OR was significantly higher in Iceland (2.11, 95% CI: 1.17; 3.82) and Norway (2.02, 95% CI: 1.04; 3.93) and, at significant level in Sweden (1.85, 95% CI: 1.00; 3.43). Non-significant OR was observed in both age groups in Denmark and Finland.

**Table 3:** **Unadjusted odds ratio (OR) of overweight in 2-17-year-old children whose parents lived separately at the time of study, stratified by age groups and country**

		2 to 9 years			10 to 17 years	
	n	OR	(95% CI)	n	OR	(95% CI)
**Denmark**	801			809		
Cohabiting		1	(reference)		1	(reference)
Separate		1.61	(0.88; 2.94)		0.83	(0.47; 1.55)
**Finland**	646			594		
Cohabiting		1	(reference)		1	(reference)
Separate		1.34	(0.73; 2.47)		1.11	(0.67; 1.82)
**Iceland**	597			666		
Cohabiting		1	(reference)		1	(reference)
Separate		2.11	(1.17; 3.82)		1.46	(0.94; 2.26)
**Norway**	599			680		
Cohabiting		1	(reference)		1	(reference)
Separate		2.02	(1.04; 3.93)		0.87	(0.54; 1.41)
**Sweden**	587			630		
Cohabiting		1	(reference)		1	(reference)
Separate		1.85	(1.00; 3.43)		1.34	(0.84; 2.13)

CI denotes confidence interval.

Cohabiting: Parents to the child lived together.

Separate: Parents to the child lived separately.

## Discussion

Across the five Nordic countries, only in Iceland was the prevalence of overweight significantly larger in children whose parents lived separately at time of study compared to children who live with both parents. Referring to our hypothesis, we would have expected a significant association between parental cohabitation and childhood overweight within each of the countries. Our results based on adjusted analyses indicated that the results were most likely caused by selection; except in Denmark, where the difference could be ascribed to differences in parental educational level and age. We found that the odds of being overweight were generally largest in children under the age of 10 years and in children whose parents lived separately.

### Comparison to other studies

Our findings are not in line with other studies specifically examining parental divorce or separation and risk of overweight in children [5, 12, 13, 17]. Three of these four studies used objective measurement of height and weight in children aged 3 to 5 [13], 5 to 14 [12], and 8 years [17]. The fourth, a cross-sectional study, used self-reported measurement in 16 to 20 years old adolescents [5]. One of the four studies, a US study of preschool children, found a statistically significant odds ratio of 1.83 for children’s overweight if the parents lived separately compared to children with cohabitating parents [13]. Another study, based on a nationally representative sample of 8-year-olds in Norway and parents’ marital status from population registry, found a 54% higher prevalence for overweight (statistically significant) and a 89% higher prevalence for abdominal obesity (statistically significant) in children whose parents were divorced compared to married parents [17]. Another one, conducted among US school children, had examined timing of family disruption and found a statistically increased risk for obesity, but not for overweight, in the two years leading up to the disruption and until 12 years after it [12]. Our study is based on self-reported height and weight and even though we found high point OR estimates in Finland, Iceland and Norway, they were not statistically significant. Furthermore, across the Nordic countries we found the greatest odds for being overweight in the youngest age group of children whose parents lived separately, although our results may be interpreted with caution as these estimates are based on unadjusted analyses. Though, the fourth study, of secular trends in overweight and obesity in Icelandic adolescents aged 16–20, demonstrated a statistically significantly increased odds of overweight and obesity in girls (odds ratio 3.36), but not among boys (odds ratio 1.71, N.S.), across the survey years 1992, 2004, 2007, and 2010 [5]. We did however not stratify our analyses by gender, so comparability to our study may not be fully warranted.

We found the largest prevalence of overweight in 2-17-years-old children living in Iceland (19.0%) and the lowest in Denmark (11.2%). This same pattern has previously been described in a review of trends in adolescent overweight in general between the Nordic countries from 1939 to 2003 [20]. The study of secular trends in overweight and obesity in Icelandic adolescents found that the probability of overweight was highest for adolescents of parents in the lowest educational strata [5]. In comparing children whose parents live separately in Denmark and Iceland in the present study, there were fewer low-educated mothers and fathers represented in Denmark than in Iceland.

Throughout the Nordic countries, the overall prevalence of overweight in children and youth was more or less the same as shown in studies examining the prevalence of overweight in children in general [3–7, 21]. Of the Nordic countries, so far only Norway and Sweden have participated in the World Health Organization (WHO) European Childhood Obesity Surveillance Initiative (COSI) [22, 23]. The pattern of difference in prevalence of overweight in Denmark and Iceland, and almost the same in Finland, Norway and Sweden, is also found in the aforementioned studies.

### Strengths and limitations

Strength in our study is that the data collection was carried out at the same time throughout the Nordic countries and the same questionnaire was used, despite a mixed-mode data collection in Denmark. This supports the comparability of the prevalence of overweight across the Nordic countries. Moreover, we observed the prevalence of overweight at two levels; overall and in children of separated parents. Furthermore, the invited study population is representative of the 2-17-year-old population in each of the Nordic countries. Our study also has some limitations. First, we cannot rule out selection bias as 41.5% of all invited participants in the 2011 NordChild survey comprised the present study population. The missing data analysis indicated that some of our results may be caused by selection effects; probably a greater proportion of high-educated parents participated. If there was a higher proportion of divorced or separated parents with overweight children among the non-responders, than we may have underestimated the prevalence proportion. Second, the prevalence of overweight is based on self-reported height and weight. It is common for parents to misclassify their overweight child as normal weight [24, 25], but we attribute this misclassification as non-differential in the present study which entails a bias towards the null (equally across each country). The fact that parents were asked to round up child’s body weight to whole kilograms is recognized and could lead to misclassification of weight status in some children. However, this error is expected to be randomly distributed and did probably not affect our findings. Furthermore, BMI is easy to calculate and widely used, but it also reflects a more lean body mass. The BMI only to a certain extent reflects the negative influence of overweight for health, mainly related to the intra-abdominal fat, and waist circumference may probably have been a more relevant anthropometric measure in this respect [26, 27]. Moreover, it remains unknown whether increase in odds of obesity exists, but it was not possible to examine due to too few cases in our sample. Third, timing of parental separation is not taken into account even though the questionnaire had asked for the age of the child when the parents were separated or divorced. Unfortunately only about 20% of the participants answered this question rendering the response rate too low for any meaningful analyses. Further, due to nature of the cross-sectional study design multiple-partner separations were also not taken into account but are of course possible. The situation of multiple divorces or separations could especially be present for the older children. In our study the majority of the children in the separate group were in the oldest age groups and if a higher prevalence of overweight children is present in multiple divorces or separations we might have overestimated this prevalence. Finally, lack of information of the major *a priory* confounder parental BMI could be another potential limitation of the study. Younger children’s risk of becoming overweight increases with parental overweight and obesity [28] and therefore we cannot rule out that our findings may be confounded by parental overweight.

### Possible explanations

The generous social service systems in the Nordic countries may be accounted for the non-difference between parental cohabitation and childhood overweight. Though, Iceland may differ from the other four Nordic countries as the prevalence of overweight in both the parental cohabiting and separate groups was greater in Iceland than in the other Nordic countries. Obesity has been shown to be associated with income inequality [29], and the ongoing financial crisis, worldwide as well as in the Nordic countries, may have been more dramatic in Iceland than the other four Nordic countries. Iceland experienced a complete collapse of its banking system in October 2008, an event that the country has not fully recovered from to date. Currently, the public in Iceland pays more out of pocket for health care and social services than ever before. This may support a reflection of increase and sudden development of inequality in Iceland.

Another possible explanation could be parental educational level as the point estimate for OR reduced when adjusting for parental educational level and age, especially in Denmark and Sweden. Interestingly, comparing the crude and fully adjusted OR models for overweight in children whose parents lived separately, the fully adjusted OR decreased but the point estimate was still high in Iceland. The fully adjusted estimate increased in Norway which partly could be explained by unmeasured confounding impact. The results in Iceland may suggest that the paternal level of education may impact childhood overweight if the parents separated in an economic insecure setting; an association found in the previous study of Icelandic adolescents [5]. Though, it may be questionable whether the Icelandic banking collapse may have caused higher rates of divorce and/or separation as the rate of 15.9% of the Icelandic parents lived separately was comparable to the other countries.

Finally, parental divorce and separation may be a stressful life transition for both adults and children, and such an episode appears to contribute to a decrease in children’s well-being [30, 31]. The impact of divorce or separation is potentially complicated, as there is multitude of potential environmental moderating factors involved in such a process [32, 33]. Further, there may be variations of interactions and events that precede and follow the divorce or separation that impact the child’s mental and physical health; i.e. decline in contact with one parent, unhealthy eating patterns, etc. [31, 34]. These factors may have been more pronounced in Iceland.

## Conclusion

Across the Nordic countries, we found no association for overweight in children whose parents lived separately compared to children whose parents lived together at time of study. Though, our finding of greater prevalence of overweight in Icelandic children whose parents live separately may indicate that the welfare society in Iceland is separating from the other Nordic countries.
